# Mesh materials and hernia repair

**DOI:** 10.1051/bmdcn/2017070316

**Published:** 2017-08-25

**Authors:** Santhini Elango, Sakthivel Perumalsamy, Krishnakumar Ramachandran, Ketankumar Vadodaria

**Affiliations:** 1 Centre of Excellence for Medical Textiles, The South India Textile Research Association (SITRA) Coimbatore - 641 014 Tamil Nadu India; 2 Cologenesis Healthcare Pvt Limited Salem - 636 140 Tamil Nadu India

**Keywords:** Hernia, Classification, Complications, Mesh materials, Recurrence

## Abstract

Hernia incidence has been observed since ancient time. Advancement in the medical textile industry came up with the variety of mesh materials to repair hernia, but none of them are without complications including recurrence of hernia. Therefore individuals once developed with the hernia could not lead a healthy and comfortable life. This drawn attention of surgeons, patients, researchers and industry to know the exact mechanism behind its development, complications and recurrence. Recent investigations highlighted the role of genetic factors and connective tissue disorders being the reason for the development of hernia apart from the abnormal pressure that is known to develop during other disease conditions. This review discusses different mesh materials, their advantages and disadvantages and their biological response after its implantation.

## 1. Introduction

Hernia is not a disease of modern society; its occurrence was noted during early 16^th^ century BC and was recognized as a surgical disease by Praxagoras of Kos because of its demand for some sort of life-saving treatment [1]. In general terms, hernia is described as a protrusion of intestine/ abdominal fat (Omentum)/ urinary bladder, commonly through a weakness or opening in the muscle wall of the abdomen. Therefore it is detected commonly in the space under the skin. The mechanism behind such opening is still under debate in the direction of anatomical defect or connective tissue disorder. Although earlier reports pointed out the role of a mechanical disparity between visceral pressure and resistance of the structures within the myopectineal orifice as the cause for hernia development, it failed to explain the factors that contribute more for its development.

From the recent investigations, it is learnt that the development of hernia is not a single event rather involving multifactorial process linking an evolutionary anatomical weakness, predisposed defects, and increased abdominal pressure. To date, the influence of each of these factors in the primary formation and recurrence of hernias is an area of significant dispute. As many number of abdominal wall hernias are reported and none of them are symptomatic but nearly all types have a potential risk of having their blood supply cut off, thereby developing severe complications when they left untreated. For example, in case of inguinal hernia, intestinal loop get trapped in the weak area of the abdominal wall, leading to closer of the intestinal channel. This further result in severe pain, vomiting, or the inability to have a bowel movement and sometimes causes *strangulation,* or restriction, of the trapped intestine’s blood supply and necessitates emergent surgery. In rare cases, strangulation of intestine considered as a life threatening since it results in death of a part of intestine. This necessitates the importance of prompt diagnosis of hernia defect to avoid its further life threatening condition [2, 3]. In India, the incidence of hernia is increasing at an alarming rate among the individuals over 50 years old and to date about 2% of the population are affected by hernia; since the data from developing countries is limited, the exact prevalence and incidence of hernia is not known.

## 2. Classification of Hernias

Evolution has clearly left human beings with a part of the abdominal wall weaker in comparison to the rest of the abdominal wall. Based on the weaker points of the abdominal wall, hernias have been classified into various types which include inguinal, umbilical, and femoral canal regions. Out of these hernias, the incidence of inguinal hernias (75%) is more compare to umbilical (9.5%), incisional (6.2%), femoral (2.7%) and other types including spigelian, hiatal, or epigastric (8.6%) [4].

## 3. Abdomen, abdominal cavity, abdominal wall and mechanism of hernia formation

The abdomen is a cylindrical chamber extending from the inferior margin of the thorax (chest) to the superior margin of the pelvis and the lower limb. The wall of abdomen contains abdominal cavity, a space that holds the abdominal organs such as stomach, small and large intestines, pancreas, liver, gallbladder, kidney and spleen. These organs are held together loosely by connecting tissues which allow them to expand and to slide against each other. All the important blood vessels including the aorta, inferior vena cava, and dozens of their smaller branches travel through the abdomen. In the front, the abdomen is protected by a thin, tough layer of tissue called fascia, which is further covered by the abdominal muscles and skin. In the rear of the abdomen are the back muscles.

Boundaries of the abdominal cavity includes the dorsal, lateral and ventral which are formed by three pairs of flat muscles (external oblique, internal oblique, and transversus abdominis) and their aponeuroses. Inside the abdominal cavity a continuous positive pressure of 2-20 mm Hg is maintained. This pressure can increase to values as high as 150 mm Hg during coughing and vomiting [5]. The abdominal wall counters this pressure, resulting in a continuous strain on the tissues of the abdominal wall. Moreover, the abdominal wall enables the body to elevate the abdominal cavity pressure during defecation, micturition and respiration.

Such increase in the intra-abdominal pressure is believed to be a contributing factor in the pathogenesis of herniation [6]. Other risk factors include obesity and chronic constipation. Sometimes hernias are thought to be the result of a single event for *e.g.* lifting a heavy object, but in fact repetitive mechanical strain is possibly the damaging factor [7]. These chronic mechanical strains, without a prior biologic defect, induce changes in structure and function of the load-bearing muscle, tendon and fascial layer. However, increased intra-abdominal pressure is speculative in nature with no clinical study to confirm its contribution to hernia formation. Furthermore, there is no adequate literature on animal models either for simulating hernia or to replicate the increased intra-abdominal pressure from erect posture gravitational forces on the floor of the abdominal wall [8]. Recent investigations, emphasized on primary fascial pathology and surgical wound failure being the fundamental biological mechanisms for herniation. In both cases, cellular and extra-cellular molecular matrix defects were noticed [9].

## 4. Role of biochemical mediators, collagen and extra-cellular matrix in hernia formation

Despite numerous predisposing factors, including anatomical features and those associated with other diseases (vomiting, coughing, obesity, chronic obstructive pulmonary disease and constipation), the underlying cause of the development of the different types of hernias is of a biologic nature. Therefore, recent day’s research targets connective tissue disorders in the process of hernia development due to its primary role in the linking of abdominal organs [10].

It is known that collagen being the principal biomechanical strength component of connective tissue provides strength and act as a scaffold in the forms of type I, II and III. Among these types, type I collagen is a matured type, forms thick collagen fibrils and provides superior mechanical strength compared to thinner type III collagen fibrils [11]. Such bulk strength for type I collagen is mainly due to the presence of intermolecular and intramolecular covalent bonds resulted from the hydroxylation and glycosylation of lysine and hydroxylysine respectively [12]. Therefore, the quality of connective tissue is significantly influenced by the quantity and ratio of Type I/III collagen synthesis and its deposition [13]. During remodeling and maturation, collagen fibers increase in diameter because of the change in the ratio of Types I and III collagen [14]. An altered Type I/III collagen ratio further result in decreased tensile strength and mechanical stability. Since, the alterations of collagen subtypes play a central role in the pathophysiology of hernias; it has become the critical component for investigation in the search for hernia genesis [15].

Battery of recent papers highlighted the presence of destructive enzymes (matrix metallo-proteinases, MMPs) and the lack of its inhibitors (Tissue Inhibitors of Metalloproteinases, TIMPS, the natural inhibitors of MMPs activity) being the cause for the altered ratio of collagen subtypes [16–18]. The main function of these enzymes is to degrade and to participate in the turnover of the extracellular matrix, by acting on certain types of collagen and elastin [19]. MMPs are proteins of a family of at least 15 zinc (Zn)-dependent endopeptidases have functions extracellularly. Out of which, MMP-1 and MMP-13 are the principle matrix enzymes responsible for fibrillar type I, II and III collagen turn over [20]. Therefore, the alterations in MMP-1 and MMP-13 protein expressions could have been the reason for the changes in the ratio of type I to type III collagen on the protein level. In line with this, over expression of MMP and immature collagen isoforms have been reported in hernia patients [11] and in patients with inguinal and incisional hernias [5, 21] respectively.

Furthermore, Klinge and coworkers [22] observed upregulated levels of MMP-1 and MMP-13 in the skin biopsies from the patients with groin hernia compared to control. In spite of this report, the same group did not find any difference in the level of MMP-1 and MMP-13 expression between excised hernial sacs of patients with inguinal hernia (direct or indirect) and controls peritoneum samples [23]. Similar results were observed when Rosch and coworkers [24] analyzed the expression of MMP-1 and MMP-13 in cultured fibroblasts from the skin of patients from primary inguinal hernia. Overall, the implications of MMP-1 and MMP-13 in hernia formation are mixed and inconclusive. Hence, the search for identifying MMPs for collagen degradation has been shifted from MMP-1 and 13 to other subtypes such as MMP-2 and MMP-9, because of its role in degrading collagen types IV and V as well as gelatin, elastin, fibronectin, and other matrix components. These MMPs (2 and 9) are derived from neutrophils and their altered expressions have been found local only to direct hernias [25]. Direct correlation between the altered expression of MMP-2 and MMP-9 with the diminished level of TIMP-1 and TIMP-2 was observed in the elderly patients [26] and inguinal hernia patients [27].

Sometimes, the defective collagen metabolisms, run in families, result in inguinal hernia and found to be the main reason for hernia recurrence [28]. This evidence highlights the genetic factors apart from MMPs, being the cause for the manifestation of disease at least in a subgroup of hernia patients. The collagen composition is of great importance because of its role in tissue remodeling apart from tissue integrity and resistance to tensile stress [29]. Recently identification of collagen gene polymorphisms (Col3A1, in type III collagen gene) in the patients with gastroesophageal reflux disease and hiatal hernias [30] further adding the importance to the collagen gene metabolism.

Findings of all these studies were similar (in spite of the involvement of different subtypes of MMPs) with respect to the downward shift in the ratio of type I/III in hernia patients. This confirms the process of herniation is not a single event and the defective collagen metabolism and family history of disease being the reason for hernia recurrence.

## 5. Hernia repair

Until 1958, the treatment for abdominal wall hernias are suture based and the major problem faced by the then surgeons were the increased recurrence of hernia [31]. To overcome this, the concept of using a mesh was introduced in 1958 by Usher. Repairs that include the use of mesh to close the defect came up with the better results but still had high recurrence rates due to the low stretching capability of the mesh/tissue complex contrasts with the highly elastic abdominal wall [32]. This resulted in shear forces at the margins of the implanted mesh, thereby the wound prior to the development of tissue integration and wound strength (first 2-3 weeks post insult) [33–35]. Owing to the significant strength of most meshes, central mesh ruptures are documented but as a rare occasion [36, 37]. In case of incisional hernias, the loss of mechanical load signaling was reported to impair fibroblast biology and the resultant collagen abnormality was found to be the cause for the recurrence [38]. In spite of all these reports, mesh repair has become the standard method in most countries and widely accepted as superior to primary suture repair. Currently, about one million meshes are used per year world-wide [39]. Therefore, surgical repair of hernia turned to be a hot area of research for keeping the recurrence rates low with few complications.

### 5.1. Meshes as biomaterials

To establish a concept of “right mesh for the right patient”, the mesh materials have been classified based on its biological response and handling characteristics. These includes non-absorbable and synthetic, non-absorbable and synthetic with a barrier, synthetic and partially absorbable, combined and biological materials.

#### 5.1.1. Non-absorbable and synthetic materials

Among the various groups, prosthetic materials falling under this category especially the polypropylene (PP), polyester (PE), and expanded polytetrafluoroethylene (ePTFE) based meshes are the one used extensively. Their biological properties with the related clinical outcomes are discussed below.

##### 5.1.1.1. Polypropylene (PP)

Polypropylene is a nonabsorbable polymer, used widely because of its high tensile strength compare to that of steel. PP is a linear aliphatic hydrocarbon with a methyl group attached to alternate carbon atoms on the chain backbone (-C_3_H_6_-). As a result, it is nonpolar in nature, highly hydrophobic, electrostatically neutral and resistant to biological degradation [40]. Currently available PP meshes are presented in both coated and uncoated forms, where the uncoated are used outside of peritoneal cavity, and the coated meshes are designed for intraperitoneal use. These materials are made in a variety of forms, either with mono or multifilament, or with a unique density and size. However, the optimal density and porosity remains unknown [41]. Both the materials are not without complications and the main disadvantage is its heavy-weight nature *(ie* the strength of PP is far greater than what is required physiologically). Therefore, abdomen is present with more foreign body and its resultant intense inflammatory response lead to side effects and complications includes formation of thick scar and contraction of the mesh. This can further aggravate the compliance problems and lead to hernia recurrence as the mesh “shrinks” (30 to 50%). This shrinkage nature of PP mesh necessitates pre-placement calculations by the surgeons to achieve a correct fit. This response can vary depending on its density, filament size, pore size, architecture, and the individual response of each carrier [42]. The clinical consequences of an intense biological response can be chronic pain, intestinal adhesions and discomfort [43, 44]. Recently light weight PP mesh has been introduced to overcome the complications of heavy-weight mesh. This mesh has been designed in such a way that decreased PP contents with much less stiffness of the abdominal wall, increased mobility, and significantly less pain (58% vs. 4%).

The overriding benefit of a PP mesh, however, is that even with its propensity to incite infection; the infections often been treated themselves without the removal of mesh. Additionally, many of the risks associated with PP are being modulated by adjusting mesh weight and porosity to promote more or less tissue in-growths. Though obviously not an inert material, PP meshes are considered to be a stable material provides an adequate service to save life.

##### 5.1.1.2. Polyester

Polyester, a multifilament mesh composed of polyethylene terephalate (PET), a heterochain linear aromatic polymer with repeating units of ester groups on either side of its ring and two ethylene moieties added to one side (-C_10_H_8_O_4_-). As such, the polymer is slightly polar, more hydrophilic, and hygroscopic than homochain hydrocarbon polymers. The mesh is available in multiple configurations for inguinal, hiatal, and incisional hernia repair. In addition, the mesh for ventral incisional hernia (VIH) repair is coated with collagen similar to PP coated mesh to prevent adhesions and can, thus, be used intraperitoneal repair. This mesh is chosen for hernia repair mainly to improve conformability and tissue in-growth with the abdominal wall [45]. Its biological response in terms of scar formation, side effects and complications are similar to PP [42]. It has been reported to degrade over time, especially during infections, therefore claiming for hernia repair [46].

##### 5.1.1.3. Expanded polytetrafluoroethylene (ePTFE)

It is not a widely used material for repairing hernias; its application is generally limited to surgical situations where visceral adhesion is of major concern. PTFE is a linear homochain polymer constructed of a carbon backbone saturated with fluorine atoms (-CF_2_-). The characteristic feature of PTFE is its inert nature, due to the presence of extreme stability of the bond between carbon and fluorine. This mesh has smaller pores sizes compared to PP, with one side large pores and the other side with a smaller pore size. As this property inhibits intestinal adhesion it also does not facilitate tissue in-growth in the abdominal wall resulting eventually in encapsulation, thus weaker hernias repair [47]. Compared to PP and PET, it exhibits minimal inflammatory reaction and comparatively lower scar density [48]. Even though minimal inflammatory reaction and lower scar density of this mesh offering for interperitonial use, this material can be broken easily. Hence the right fixation is quite important [49]. The complications associated with both polyester and PTFE meshes from the clinical uses have exposed a need for a better mesh material.

#### 5.1.2. Non-absorbable and synthetic with a barrier

Prosthesis with either an absorbable or a nonabsorbable barrier is used for preventing bowel adhesions when it placed intraperitoneally. This mesh is engineered in such a way that barrier on one of their faces to facilitate direct contact with the viscera. Here the barrier minimize the biological response, provide the limited opportunity for initial adhesion to the material thereby reducing the activation of imflammatory cytokines and cells, ultimately inhibit the onset of inflammatory cascade. Selection of an optimum size and its proper fixation are mandatory. The possible barriers are ePTFE, polyurethane, oxidised regenerated cellulose, omega-3 fatty acids, collagen, or beta glucan. Numerous experimental studies show the anti-adhesive properties of these compounds, both with physical (non-absorbable) or chemical (absorbable) barriers [50–54]. On the other hand, the literature is scarce regarding their observed clinical behaviour in reoperations.

#### 5.1.3. Synthetic and partially-absorbable meshes

The purpose of constructing partially absorbable mesh is mainly to reduce the density of the biomaterial and its subsequent inflammatory reaction while maintaining the intraoperative handling characteristics and long-term wound strength. Currently available meshes are developed with a fusion of non-absorbable (PP) and absorbable materials for eg polyglactin 910 and poliglecaprone 25 [55]. The most common and extensively studied materials are poly (L-lactide-co-glycolide) and PP, engineered in a combination of 10/90 respectively. Polyglycolide copolymer is linear, aliphatic polyester with a single ester and ethylene group (-CH_2_COO-) and somewhat hydrophilic in nature therefore allowing for hydrolysis of ester group. Usage of this kind of mesh material has been known to cause less fibrosis and structural changes which further results in larger pores, and less chronic inflammation [56]. But some reports identified differences in the variety of inflammatory markers and its biological response upon using this mesh when compared to non absorbable compound such as PP [57, 58]. In spite of the evidence for deterioration of hernia, various clinical studies illustrated less pain and discomfort upon usage of these prostheses.

#### 5.1.4. Combined Meshes

The main purpose of combined mesh material is to prevent the complications by taking advantages of the best traits from 2 different meshes. In case of polyester and PTFE combined meshes, former allows the abdominal wall tissue in-growth whereas later prevent the occurrence of intestinal adhesion achieved through different pore size of the mesh. Manufactures are developing various combination mesh materials in an attempt to provide surgeons with an improved synthetic mesh. A more recent movement in the design of combination synthetic meshes is to construct a mesh consisting of a PP or PET base coated with absorbable polymers. It is reported that, adhesion of intestine with hernia meshes usually occurs within a week of the initial surgery [44]. Thereafter, a layer of peritoneal cells coat the mesh and prevent the further risk of adhesion formation [59]. From this finding, it is found that synthetic meshes only need a temporary adhesion barrier, hence the use of absorbable polymer coatings. Conflicting results, however, do not provide enough corroborative data to currently determine which type of combination mesh performs the best in the clinical setting [60].

Yet another movement in the development of synthetic hernia repair meshes is to create materials consisting of 100% absorbable materials. Currently, available clinical data are not sufficient enough to determine the viability of absorbable meshes. But the advantage of absorbable meshes over others is their use in contaminated fields, since it provides benefit of non-removal therefore can be placed in direct contact with the bowel [44]. Another potential benefit of absorbable meshes is the formation of a collagen layer upon healing. The preliminary studies using absorbable polymer polyglactin 910, showed the recurrence hernia and catastrophic failure when the collagen layer is been replaced which is not strong enough to prevent the above mentioned complications [61]. Also, it is extremely important to achieve polymer degradation rates that are in sync with tissue in-growth. Although this material is not promising as a stand-alone material, the ability of this material to remodel tissue may lead into a novel direction for hernia repair materials.

#### 5.1.5. Biological meshes

The primary importance for the construction of biological mesh is to overcome the problems of synthetic meshes and to provide mechanical support, tissue remodeling along the mesh scaffold in order to create highly organized collagen network thereby to establish new vascular access to the hernia site.

Even though currently available biologic meshes are seemed to have different origin, these are all common in taking collagen rich tissues from human or animals, stripping of all cellular contents and stabilizing the resultant extracellular protein structure to act as a collagen scaffold for the in growth and deposition of fibroblast and collagen respectively [62]. Removal of cellular contents also offers an advantage of impeded inflammatory response and immune-mediated rejection of the implanted material and leaves a number of beneficial components includes a complex agglomeration of structural and functional proteins, glycosamino-glycans, glycoproteins, and numerous other small molecules and growth factors [63]. These residual components give the decellularized extracellular matrix (ECM) the unique property of modulating a wound healing response instead of facilitating scar tissue formation [64]. Most of the research on these materials is from difficult clinical situations. Because these materials induce angiogenesis for the remodeling of the tissue, potentially resist infection [65, 66], and they have a moderately good success rate for salvaging contaminated and infected fields, especially when placed with wide overlap. Other findings demonstrate some resistance to adhesion formation [67]. Therefore, the basic concept behind the development of these types of materials is that, they provide proper environment for the population of native cells, generation of connective tissue which ultimately lead to the replacement of defective tissue present in the hernia defect.

Although biological meshes show great promise in repairing hernia, currently surgeons are hesitant to opt for this over synthetic mesh materials. Because, the connective tissue formed by these materials is only 70-80% strong, emphasizing the inherent defect of their native tissue. Therefore, there is a greater chance for the recurrence of hernia upon usage of these meshes. Additionally, some surgeons remain skeptical of biologic meshes due to reports of higher mechanical failure compared with synthetic meshes. Also, there looms the possibility of disease transmission with biologics meshes, however, no reports on disease transmission are currently published [62]. With a theoretically increased risk of long-term recurrence, relatively high cost, and no clear benefit, eventually quelled with more advanced research and clinical trials.

## 6. Biological responses upon insertion of a mesh

Several biologically-active factors are released by the different cells and affect the biological response to the mesh material. Primary response is the formation of a layer of palmitic proteins such as albumin, IgG and fibrinogen around the mesh material immediately after implantation. These proteins are known to have interaction with the cellular components viz platelets, monocytes, macrophages, and polymorphonuclear leukocytes involved in the inflammatory response [68]. Since the concentration of proteins adsorbed depends on the type of prosthetic material, their interaction is also different. Upon adsorption, the surface activates the classic and alternative complement pathways, especially generating factor C5a (a chemotactic factor for inflammatory cells). Activation of these compliment pathways are again depends on the type of mesh materials.

In continuation with this, growth factors such as platelet derived growth factor (PDGF, support the smooth muscle cells and fibroblasts proliferation), fibroblast growth factor (FGF, a potent mitogen for smooth muscle cells, endothelial cells and fibroblasts), transforming growth factor β (TGF β, promotes the production of fibroblasts and activates monocytes), insulin-like growth factor (IGF, a potent chemotactic for endothelial cells produced by platelets and fibroblasts) and epidemic growth factor (EGF, promotes the production of extracellular matrix proteins, high concentrations in platelets) are activated and playing a significant role in the repair of hernia [69]. Expression of FGF [70] and TGF β [71] increases in the presence of mesh materials.

Around a week after implanting the mesh material, the population of mononuclear phagocytic cells differentiates into macrophages. These cells secrete a wide number of effectors which help to modulate the biological response [72]. The inflammatory reaction seals the foreign body in an epithelioid granuloma. In the presence of indigestible prosthetic material, the macrophages coalesce into foreign-body giant cells [73]. The role of these cells is not clear, but they stay indefinitely in the presence of a nonabsorbable prosthesis.

The final stage of the biological response is the synthesis of connective tissue. Primarily the collagen is synthesized and excreted by fibroblasts as monomeric form into the extracellular space where it polymerises into an insoluble helicoidal structure. A collagen network is produced for around 21 days, then there is an alteration in the ratio of collagen type III and I *ie* there will be a reduction in the level of immature collagen (type III) and raise in the mature collagen (type I). The three-dimensional collagen network grows around and through the prosthesis. As a consequence of this remodelling, its mechanical strength increases progressively until~6 months after performing the surgical wound. However, at the end of this period, the newly formed tissue only has 80% of the normal mechanical strength of the skin or fascia. Other properties, such as its elasticity or energy absorption capacity will be even lower. The final result is a weaker and more fragile tissue than normal. A non-absorbable prosthesis covered by the newly-formed tissue will help to improve these weaknesses (74, Table 1).

**Table 1:** Mesh materials and their biological response.

S.No	Material	Commercially available meshes	Inflam mation	Granu locytes	Fibro blasts	Giant cells	Macropha ges	Adhesions	Classi fication	Tissue in growth	Resis tance to infection
1.		Marlex	Abun dant^75^	Moderate	Moderate^75^	Moderate^75^	Moderate	Moderate^76^	permanent^76^	Extensive^76^	High^76^
2.	Polypro pylene	Prolene	Moderate^77^	Slight^77^	Abundant^77^	Moderate^77^	Mild to Moderate^77^	Mild to Moderate^76^	permanent^76^	Extensive^76^	High^76^
3.											
4.	Polyester	Mersilene	Abundant^75^	Abundant^75^	Abundant^75^	Abundant^75^	Abundant^75^	Mode rate	permanent^76^	Exten sive	May promote
5.	Expanded polytetra-fluroethy-lene	Gore-tex	Moderate^75^	Moderate^75^	Moderate^75^	Moderate^75^	Moderate^75^	Rare	Perma nent	Mini mal	May promote
6.		Intramesh T1	Minimal^80^	Mild to Moderate^81^	Moderate	Moderate^81^	Moderate^81^	Mild to Moderate^81^	Permanent^81^	Allows on PP side	Nil^81^
Synthetic and partially-absorbable meshes											
7.	Polyglactin 910	Vicryl	Nil^76^	_Nil_^76^	Minimal^76^	_Nil_^76^	_Nil_^76^	Rare	Partially-absorbable^86^	Mild Moderate	Mesh dissolves
8.	Poligleca-prone 25	Ultrapro®	Moderate^79^	Moderate^79^	Abundant to slight	Slight/ Moderate to Moderate	Slight/ Moderate to Moderate	Mild to Mode rate	Partially-absorbable^86^	_Nil_^79^	_Nil_^80^
9.	Polypro pylene	C-Qur®	Slight to Moderate	Moderate^82^	Slight/ Moderate to Moderate	Slight/ Moderate to slight	Slight/ Moderate to slight	Less	Absorbable^79^	_Nil_^79^	_Nil_^80^
10.	Polyester	Parietex composite	Moderate^79^	Moderate^79^	Abundant^79^	Abundant^79^	Abundant^79^	Mild80 to Moderate^79^	Absorbable^79^	High^80^	_Nil_^80^
11.	Composite meshes	Vypro II	Mode rate	Moderate	Mild to Moderate	Mild to Moderate	Moderate	Mini mal	Partially	Moderate	Nil
12.		Proceed	Moderate^77^	Slight^77^	Abundant^77^	Moderate^77^	Mild to Moderate^77^	High^78^	Absorbable^79^	Allows on PP side^80^	Nil
13.	Human -Derived	AlloDerm®	Moderate to nil^84^	Moderate^84^	Moderate to Minimal^84^	Moderate to minimal^84^	Moderate^84^	Nil^84^	Partially-absorbable^84^	High^84^	Nil^84^
14.	Non Human derived	CollaMend	Moderate^84^	Moderate^84^	Moderate to Minimal^84^	Minimal to moderate^84^	Minimal to moderate^84^	_Nil_^84^	Partially-absorbable^84^	High^84^	_Nil_^84^

This clearly indicates the intensity and duration of all this host/prosthesis reaction is depend on the type and quantity of the material being used. In turn, this affects the following factors: the greater or lesser rigidity of the abdominal wall after the operation, the long-term pain or the sensation of noticing the material in the area of the surgery, and the greater or lesser contraction of the prosthesis/tissue with its possible influence on recurrence [68].

## 7. Selection of a mesh material

Before selecting the material, it is important to know the characteristics of a mesh in terms of the material to be replaced and strengthened during the insertion of a mesh into the abdomen. However, the abdominal wall develops strength (16 N/cm) when it is subjected to intra abdominal pressure and develops elasticity as well. The mean elasticity of the wall of a male and female at 16 N/cm is found to be around 23 [85] % in the vertical direction and 15 [86] % in the horizontal direction. In spite of this strength and elasticity, the abdomen has the capacity to withstand the pressure of up to 252 mm Hg during coughing, jumping and lifting weights. To handle such high pressure, the abdomen increases its strength up to 27 N/cm [58]. Therefore bearing these values of between 16 and 27 N/cm in mind they can be related to the force necessary to break the prostheses which are normally used to repair hernias.

Few reports confirmed the detachment of mesh material at the edge of the hernia defect when the intra abdominal pressures increases up to 200 mm Hg except when the prosthesis has a wide margin (at least 4 cm) with regard to the edge. However, incidence of this event is observed more frequently when the elastic direction of the mesh is placed disparate to the direction of defect’s longest diameter [87].

Recent advancements came up with the large number of different varieties of mesh material for the repair of hernia. In spite of this, surgeons still using PP material because of its rigidity and comfort. After implantation of this material, the resultant complications are very severe and result in the recurrence of hernia. Therefore, before choosing the material for a particular hernia defect, it is better to look for the properties of a mesh (Table 2) for a given case. Prosthesis used for hernia repairs can be of any type, non-absorbable, composite (combination of absorbable and non-absorbable fibres) or with an absorbable or a non-absorbable barrier. For intra-abdominal placements, any mesh that will prevent bowel adhesions should be used. It can be either ePTFE surgical mesh or any one of the newly engineered meshes with an absorbable or a nonabsorbable barrier. Non-absorbable or composite mesh is recommended for hernia repair where it will not come in contact with the bowel. Prosthesis with a barrier should be used only for intra-abdominal placement to prevent bowel adhesions since it is increasingly difficult to defend the use of a biomaterial that has no adhesion barriers [33].

**Table 2:** Characteristics of an ideal mesh.

S.No	Characteristics
1.	Resistance
2.	Durability
3.	Tissue tolerance
4.	Flexibility and memory
5.	Non-migration
6.	Stability
7.	Pervious pores
8.	Sterilizability
9.	Non-carcinogenic
10.	Should block infectious diseases transmission
11.	Resist shrinkage
12.	Should have the ability to degrade itself over time and be easy to manufacture
13.	Minimal adhesion formation
14.	Should bear excellent tissue in growth with minimal shrinkage
15.	Should allow the formation of seroma but not fistula
16.	Promote minimal pain
17.	Should not change compliance of abdominal wall
18.	Should have elasticity in more than one dimension
19.	Should possess the quality of adhesiveness

Next important matter to consider is the size of the mesh. It must be at least 15 x 15 cm for an inguinal hernia. For repair of umbilical, ventral and incisional hernia, it should be at least 4 cm wider than the defect. It is better to initially measure the size of the defect with the scale and then select a mesh of appropriate size. It should be wide enough to cover the defect in all directions since a smaller size may lead to protrusion of the mesh into the defect and result in a recurrence [33].

## 8. Summary

From the various studies, it is found that the hernia is not a single event rather involving multiple processes and the treatment depends completely on the mesh materials. Recent days, surgeons are provided with the sufficient number of mesh materials for a given hernia case. Therefore acquiring knowledge about these materials will help surgeons as well as patients to provide a better treatment in terms of reduced mesh associated complications and recurrence rate.
